# Reproducibility of a battery of human evoked pain models to detect pharmacological effects of analgesic drugs

**DOI:** 10.1002/ejp.1379

**Published:** 2019-04-05

**Authors:** Pieter S. Siebenga, Guido van Amerongen, Pieter Okkerse, William S. Denney, Pinky Dua, Richard P. Butt, Justin L. Hay, Geert J. Groeneveld

**Affiliations:** ^1^ Centre for Human Drug Research Leiden The Netherlands; ^2^ Pfizer Inc. Cambridge Massachusetts; ^3^ Pfizer Ltd. Cambridge UK; ^4^ Leiden University Medical Center Leiden The Netherlands; ^5^ Present address: Human Predictions LLC Cambridge Massachusetts

## Abstract

**Background:**

Although reproducibility is considered essential for any method used in scientific research, it is investigated only rarely; thus, strikingly little has been published regarding the reproducibility of evoked pain models involving human subjects. Here, we studied the reproducibility of a battery of evoked pain models for demonstrating the analgesic effects of two analgesic compounds.

**Methods:**

A total of 81 healthy subjects participated in four studies involving a battery of evoked pain tests in which mechanical, thermal and electrical stimuli were used to measure pain detection and tolerance thresholds. Pharmacodynamic outcome variables were analysed using a mixed model analysis of variance, and a coefficient of variation was calculated by dividing the standard deviation by the least squares means.

**Results:**

A total of 76 subjects completed the studies. After being administered pregabalin, the subjects’ pain tolerance thresholds in the cold pressor and pressure stimulation tests were significantly increased compared to the placebo group. Moreover, the heat pain detection threshold in UVB‐irradiated skin was significantly increased in subjects who were administered ibuprofen compared to the placebo group. Variation among all evoked pain tests ranged from 2.2% to 30.6%.

**Conclusions:**

Four studies using a similar design showed reproducibility with respect to the included evoked pain models. The relatively high consistency and reproducibility of two analgesics at doses known to be effective in treating clinically relevant pain supports the validity of using this pain test battery to investigate the analgesic activity and determine the active dosage of putative analgesic compounds in early clinical development.

**Significance:**

The consistency and reproducibility of measuring the profile of an analgesic at clinically relevant doses illustrates that this pain test battery is a valid tool for demonstrating the analgesic activity of a test compound and for determining the optimal active dose in early clinical drug development.

## 1 INTRODUCTION

Demonstrating the analgesic potential of a new analgesic compound in pain patients can be inherently difficult, particularly in the presence of other symptoms such as fever and/or malaise. Moreover, pain can occur in patients with clinical conditions and diseases that are usually treated with therapeutic interventions that can alter the perception of pain, for example by inducing side effects such as sedation (Drewes, Gregersen, & Arendt‐Nielsen, 2003). An alternative to studying new putative analgesic compounds in patients with pain is to assess their analgesic potential in healthy subjects using an evoked pain model. Over the years, a variety of pain models has been developed for measuring pain thresholds (Andersen et al., 1996; Bishop, Ballard, Holmes, Young, & McMahon, 2009; Brennum, Kjeldsen, Jensen, & Jensen, 1989; Dahan et al., 2004; Drewes, Petersen, Qvist, Nielsen, & Arendt‐Nielsen, 1999; Eckhardt et al., 1998; Hay, Okkerse, Amerongen, & Groeneveld, 2016; Jones, McQuay, Moore, & Hand, 1988; Olesen, Andresen, Staahl, & Drewes, 2012; Olofsen et al., 2005; Petersen‐Felix et al., 1995; Polianskis, Graven‐Nielsen, & Arendt‐Nielsen, 2001, 2002; Schilder, Magerl, Hoheisel, Klein, & Treede, 2016; Siebenga et al., 2018). Historically, these models have been used as a single test; however, based on studies measuring the effect of analgesic compounds on evoked pain it has become clear that some drugs can yield significant results in one pain model but can fail to have an analgesic effect when using a different pain model (van Amerongen, Boer, Groeneveld, & Hay, 2016; Arendt‐Nielsen, Curatolo, & Drewes, 2007; Brennum et al., 1994). This inconsistency is due—at least in part—to the wide variety of pain signalling mechanisms and pathways at the peripheral and spinal levels, which are sensitive to different analgesics. Therefore, when using only one evoked pain model, selecting the correct model is essential. For example, using a single test can increase the likelihood of obtaining a false‐negative result, and decisions based on that result are not only costly, but could also lead to the incorrect decision to terminate the development of a potentially active analgesic compound. To minimize this possibility, the pharmacological mechanism of action of a compound should be assessed using a battery of pain models, thereby increasing the likelihood of accurately measuring nociceptive activity and helping predict the optimal dosing range for putative analgesic compounds.

A battery of evoked pain models involving human subjects provides the opportunity to use several pain models in the same study and allows the researcher to profile the analgesic in an early phase of clinical development. For example, such a battery can help determine whether the compound acts centrally or peripherally, the modality of pain for which the compound is best suited (e.g., nociceptive, neuropathic or inflammatory pain), and whether additional effects such as sedation and/or tolerance contribute to the compound's mode of action (Lötsch et al., 2016; Oertel & Lötsch, 2013; Okkerse et al., 2017; Olesen et al., 2012; Staahl, Olesen, Andresen, Arendt‐Nielsen, & Drewes, 2009; Vollert et al., 2018). Moreover, results obtained using a battery of pain models can provide important information regarding dose finding and can provide proof‐of‐analgesia for new analgesic compounds (Arendt‐Nielsen, Curatolo et al., 2007; Arendt‐Nielsen, Frøkjaer et al., 2007; Okkerse et al., 2017).

The ability to consistently obtain reproducible results is an essential attribute of pain models, particularly in the early stages of clinical drug development. Unfortunately, however, reproducibility can be overshadowed by publication bias, in which innovation and/or the strength of the study's findings serve as the primary basis for the submission and ultimate acceptance of a research article. Reproducibility is an important feature in science, as it can be used to verify prior results, to clarify whether results can be generalized to a wider population or other populations, and/or to test the hypotheses proposed in the initial study (Fabry & Fisher, 2015). Before proper conclusions can be drawn from a study, unknown factors and dependent variables should be minimized, and comparability between studies should be optimized, as inconsistencies and/or contrasting results between studies can be confusing and can lead to the wrong—or even a potentially dangerous—conclusions (Prinz, Schlange, & Asadullah, 2011). In a recent study entitled “The Reproducibility Project,” investigators attempted to reproduce the methods in 100 studies published in top psychology journals, but concluded that only 39% of the trials could be reproduced unambiguously (Open Science Collaboration, 2015). Although this project was based on psychology studies, a similar discussion in the field of biomedical research has recently emerged, with similar outcomes regarding a lack of reproducibility of the results in the original publications (Baker, 2016; Begley & Ioannidis, 2015; Eglen et al., 2017; Fabry & Fisher, 2015; Munafò et al., 2017). Despite its underlying message, no clear conclusions can be drawn from The Reproducibility Project, as no single study can provide a definitive outcome; rather, it is the cumulative process that increases the reliability of a method. Staahl and colleagues concluded that their models are generally reproducible, but noted that overall variation can be high in certain cases due to a variety of factors, including stimulation duration, the site of stimulation, and the age and gender of the subjects (Staahl, Reddy, Andersen, Arendt‐Nielsen, & Drewes, 2006). High variability is not unusual when using evoked pain models with human subjects (Rollman & Harris, 1987; Taylor, McGillis, & Greenspan, 1993), as differences in the subjects’ pain perception can cause high inter‐subject variability. Moreover, using a study design that is appropriate to each study compound is particularly important.

Here, we compared the results of four recent studies that used an identical battery of evoked pain models in healthy subjects; specifically, these studies used a nearly identical study design as well as identical drugs and doses. The aim of our study was to examine the reproducibility of using a battery of pain models, including thermal, electrical and mechanical stimuli, to profile the analgesic effects of two commonly used analgesics.

## 2 METHODS

### 2.1 Design

All four studies had a randomized, double‐blind, placebo‐controlled, cross‐over single‐dose design and were registered with EudraCT (2013‐003443‐28, 2014‐003015‐12, 2014‐003553‐34 and 2014‐004468‐39, referred to hereafter as studies I through IV, respectively); in addition, studies II–IV were also registered at ClinicalTrials.gov (NCT02238717, NCT02260947 and NCT02349607). The studies were designed to investigate the ability of new and established analgesics to induce analgesia in healthy subjects and used a battery of pain models. Subjects participated in up to three phases: (a) a screening/training phase; (b) four daily in‐house periods (for studies I and II) or five daily in‐house periods (for studies III and IV) at 7‐day intervals; and (c) a post‐treatment (follow‐up) call scheduled 6–8 days after the last study drug administration (for study I) or 7–10 days after the last study drug administration (studies II–IV). At screening, each subject underwent a complete medical screen as well as the battery of pain tests. In addition, during the screening phase, each subject's minimal erythema dose (MED) was determined 24 ± 2 hr after applying six ascending intensities of ultraviolet B (UVB) irradiation to the subject's skin.

Each in‐house period started on day 1 with exposure to UVB at three times the subject's MED (3 × MED). After an overnight stay in the research unit, the subject's baseline pharmacodynamics (PD) profile was measured twice 24 ± 2 hr after UVB irradiation. A baseline blood sample for pharmacokinetics (PK) analysis was drawn before dosing, and PD and PK were assessed 0.5, 1, 2, 3, 4, 6, 8 and 10 hr after dosing.

All trials were conducted at the clinical research unit of the Centre for Human Drug Research in Leiden, the Netherlands. Each subject received the study treatment regimen based on his/her randomized assignment. Both the subjects and the investigators were blinded with respect to the treatment regimen. The studies were conducted in accordance with the Declaration of Helsinki and its amendments and in accordance with established guidelines for Good Clinical Practice. All protocols were approved by a Medical Ethics Committee (MEC); study I was approved by the MEC of Leiden University Medical Center (Leiden, the Netherlands), and studies II–IV were approved by the Stichting Beoordeling Ethiek Biomedisch Onderzoek (Assen, the Netherlands).

### 2.2 Subjects

A total of 81 healthy subjects enrolled in the four studies; three subjects participated in both study II and study IV. Studies II, III and IV had identical inclusion and exclusion criteria, whereas study I used slightly different criteria.

Females were allowed to participate in study I but were not included in studies II–IV. The age range for participating in study I was 18–45 years; the age range for participating in studies II–IV was 18–55 years. For participation in study I, the allowed range for BMI was 18–30 kg/m^2^; for studies II–IV, the allowed range for BMI was 17.5–30.5 kg/m^2^. Finally, for study I the allowed values for systolic and diastolic blood pressure were 100–160 and 50–95 mmHg, respectively; for studies II–IV, the allowed values for systolic and diastolic blood pressure were ≥50 and ≤95 mmHg, respectively.

For all studies, subjects were excluded if they had evidence of clinically relevant findings while taking the medical history and/or during the physical examination, or evidence of clinically significant abnormalities with respect to the subject's ECG, vital signs, blood chemistry, and/or haematology results. In all four studies, only healthy subjects with a Fitzpatrick skin type of IV or lower were included. Additional exclusion criteria included widespread acne, tattoos and/or scarring on the back, any clinically significant medical condition (particularly any existing condition that would affect the subject's sensitivity to cold), and subjects who indicated an intolerability for nociceptive tests at screening or who achieved tolerance at >80% of maximum input intensity for any nociceptive test involving cold, pressure, heat or electrical stimuli. Other exclusion criteria included a positive drug test or urine‐based pregnancy test (for female subjects, relevant only to study I), hypersensitivity to the study treatments and the use of prescription and/or non‐prescription drugs or dietary supplements within 7 days or 5× the half‐life (whichever was longer) prior to the first study dose. Finally, subjects were instructed to avoid excessive exercise, dietary restrictions, alcohol, nicotine and caffeine for 24 hr prior to dosing and while at the clinical research unit.

All participating subjects provided written informed consent.

### 2.3 Treatment

Pregabalin (300 mg) was administered to the subjects in all four studies, and ibuprofen (600 mg) was administered to the subjects in studies I, III and IV. For study I, pregabalin was supplied by Pfizer and ibuprofen was provided by Reckitt Benckiser Healthcare (Hoofddorp, the Netherlands); for studies II–IV, pregabalin and ibuprofen (study II excluded) were supplied by Pfizer. The Pharmacy Department at Leiden University Medical Center prepared the study compounds together with identical placebos. To ensure blinding of the participants and researchers, a double‐dummy design was used in studies II, III and IV; in study I, all study drugs were over‐encapsulated in order to render them indistinguishable from each other. All compounds and placebos were taken orally with 240 ml of water.

### 2.4 Pharmacodynamics

Pharmacodynamics was measured using an integrated battery of pain models designed to measure various modalities of pain. These models have been described previously (Hay et al., 2016), and all assessments were performed by trained personnel. In all four studies, tasks were performed in order to measure the pain detection threshold (PDT) and the pain tolerance threshold (PTT). For each pain model (with the exception of the heat pain model), pain intensity was measured continuously, and the subject rated their pain intensity using a 100‐mm electronic visual analogue scale (eVAS), with 1 and 100 mm defined as PDT and PTT, respectively. The testing equipment automatically terminated the pain assessment when PTT was reached or when the safest maximum stimulation was applied, whichever occurred first. Electrical PTT, pressure PTT, cold pressor PTT and heat pain PDT were the primary endpoints of interest.

#### 2.4.1 Electrical stimulation

This method was adapted from the protocol reported by Olofsen and colleagues (Dahan et al., 2004; Olofsen et al., 2005). For inducing cutaneous electrical pain, two Ag‐AgCl electrodes were placed on a clean patch of skin overlying the left tibia; specifically, the centre of the first electrode was placed 100 mm distal from the caudal end of the patella, and the centre of the second electrode was placed 135 mm below the first electrode. Electrical resistance between the electrodes was verified as <2 kΩ.

Each stimulus (a 10‐Hz tetanic pulse lasting 0.2 ms) was administered by a computer‐controlled constant current stimulator. Current intensity began at 0 mA and increased in 0.5‐mA increments every second; the maximum current intensity was 50 mA. Pain intensity after each stimulation was measured using the eVAS, and the stimuli continued until the subject's pain tolerance level was reached or until a maximum of 50 mA was applied, whichever occurred first (Dahan et al., 2004; Olofsen et al., 2005).

#### 2.4.2 Pressure stimulation

This method was adapted from the protocol reported by Polianskis and colleagues (Polianskis, Graven‐Nielsen, & Arendt‐Nielsen, 2001, 2002). An 11‐cm‐wide tourniquet cuff (VBM Medizintechnik GmbH, Sulz am Neckar, Germany) was placed over the gastrocnemius muscle, and the pressure applied was increased linearly by 0.5 kPa/s under the control of a model ITV1030‐31F2N3‐Q electro‐pneumatic regulator (SMC Corporation, Tokyo, Japan) driven by a Power1401 analogue‐to‐digital converter and Spike2 software (Cambridge Electronic Design, Ltd., Cambridge, UK). During the test, the subject was seated comfortably with their feet flat on the floor; pain intensity was rated using the eVAS, and the pneumatic pressure was increased until the subject indicated his/her PTT or until a maximum pressure of 100 kPa was applied (whichever occurred first), at which point the device released the pressure on the tourniquet.

#### 2.4.3 Cold pressor

The method for applying cold pressor pain was based on the methods reported by Eckhardt and colleagues (Eckhardt et al., 1998) and Jones and colleagues (Jones et al., 1988). During the test, the subject placed his/her non‐dominant hand in a thermostat‐controlled water bath (LAUDA, Germany) at 35 ± 0.5°C (minimal depth: 200 mm) for 2 min. After 1 min and 45 s, a blood pressure cuff on the upper arm was inflated to 20 mmHg below the subject's resting diastolic pressure. At 2 min, the subject was instructed to remove the hand from the warm water bath and place the same hand immediately into a similar sized water bath at 1.0 ± 0.5°C. The subject was then instructed to indicate when the PDT was reached (based on the initial change in sensation from cold and non‐painful to painful) and to record the increase in pain intensity by moving the eVAS slider. When the subject's pain tolerance was reached (i.e., the sensation was no longer tolerable, defined as 100 mm on the eVAS) or after 120 s (whichever occurred first), the subject was instructed to remove his/her hand from the cold water bath, at which point the blood pressure cuff deflated.

#### 2.4.4 Heat pain and inflammatory heat pain

Heat pain was assessed using a method adapted from Bishop and colleagues (Bishop et al., 2009). At the screening visit, UVB irradiation was applied using a TL01 narrow‐band UV lamp (Phillips, the Netherlands) in ascending doses (corresponding to different durations of irradiation) at six separate 1 cm × 1 cm patches of skin on the subject's upper back in order to determine that subject's MED, defined as the minimum UVB dose that produced the first clearly discernible erythema.

For each subject, a 3 cm × 3 cm patch of skin on the back was exposed to 3 times the individual MED of UVB 24 ± 2 hr prior to the first battery of tasks; this irradiation was applied to the subject's back in order to produce a homogeneous patch of skin with erythema and hyperalgesia.

A 3 cm × 3 cm thermode (Medoc, Ramat Yishai, Israel) was used to measure the thermal PDT using an initial temperature of 34°C, which was increased at a rate of 0.5°C/s. First, the average PDT from three stimuli was measured on a non‐irradiated (normal) patch of skin (contralateral to the site of UVB irradiation), followed by the same measurements on the UVB‐irradiated skin.

### 2.5 Sampling for pharmacokinetics

Throughout each study, blood samples (8 ml) were collected from each subject in order to provide the minimum volume of 4 ml plasma required for PK analysis. Blood samples were collected in tubes containing K_2_‐EDTA. Plasma concentration was measured over time, and for each subject, the maximum concentration (*C*_max_), the area under the concentration–time curve (AUC) from time 0 to the last sample above the limit of quantification (AUC_last_), the AUC from time 0 extrapolated to time infinity (AUC_inf_), the time of *C*_max_ (*T*_max_) and the half‐life (*T*_1/2_) were calculated for both pregabalin and ibuprofen, where applicable.

### 2.6 Statistical analyses

The primary comparison was the mean effect of pregabalin compared to placebo up to 5 hr in study I and up to 6 hr in studies II–IV. The mean effect of ibuprofen compared to placebo was analysed up to 4 hr in all four studies. In each study, the least squares means (LSM) and standard deviation (*SD*) were analysed using a mixed model analysis of variance, with treatment, time, and treatment by time as fixed factors and subject, subject by treatment, and subject by time as random factors; the average baseline measurement was used as a covariate. Different times for the duration of effect were selected based on the half‐life of the respective drug.

For each pain model, reproducibility across subjects was evaluated for both pregabalin and ibuprofen using the coefficient of variation (CV). The CV expressing the inter‐subject variability was calculated by dividing the *SD* by the LSM and is expressed as a percentage. The confidence interval (CI) reported for study I is the 95% CI; the CI in studies II–IV is the 90% CI. In studies II–IV, the confidence intervals of the heat pain assessments (in UVB‐irradiated and non‐irradiated skin) were back‐transformed.

## 3 RESULTS

A total of 81 healthy subjects (eight women and 73 men) satisfied the inclusion and exclusion criteria and were randomized to receive analgesic or placebo. All 81 subjects received pregabalin, and 61 subjects (eight women and 53 men) received ibuprofen. The demographics of the subjects were similar between all four studies and are summarized in Table 1. Of the 81 subjects who enrolled in the study, 76 completed their respective studies and were included in the final analyses. In both studies I and III, one subject dropped out; in study IV, two subjects dropped out; finally, one subject withdrew from study III due to abnormalities on ECG. No study drug‐related reasons were cited by the subjects who dropped out, and the ECG abnormality that caused the subject in study III to withdraw was not deemed to be related to the study drug.

**Table 1:** Participant characteristics

Study	I (*n* = 16)	II (*n* = 20)	III (*n* = 20)	IV (*n* = 25)
Age, years	21.9 (19–25)	31.8 (18–50)	26.0 (18–43)	26.9 (18–46)
BMI, kg/m^2^	21.9 (19.4–25.1)	23.3 (18.5–27.2)	23.7 (18.2–29.8)	23.0 (18.5–27.0)
Male/Female	8/8	20/0	20/0	25/0
Fitzpatrick skin type				
Type I	2 (12.50%)	0	0	0
Type II	2 (12.50%)	4 (20.00%)	1 (5.00%)	6 (24.00%)
Type III	9 (56.25%)	11 (55.00%)	12 (60.00%)	13 (52.00%)
Type IV	3 (18.75%)	5 (25.00%)	7 (35.00%)	6 (24.00%)

Note

BMI, body mass index. Age and BMI are presented as the mean (range).

A summary of the CV values, *p*‐values and LSM effect versus placebo (primary outcomes) is presented in Table 2. In all four studies, subjects who received pregabalin had a significantly higher PTT in the cold pressor test compared to subjects who received placebo. Specifically, the LSM (estimate of the difference) in studies I, II, III and IV was 25.1 s (46.4%), 23.3 s (29.7%), 32.4 s (21.4%) and 21.0 s (22.4%), respectively. Moreover, compared to placebo pregabalin also had a significant effect on PTT in the pressure stimulation test and on PDT in the heat pain assessment of the non‐irradiated area. For the pressure stimulation test, the LSM (estimate of the difference) in studies I, II, III and IV was 47.6 kPa (14.1%), 43.7 kPa (12.1%), 57.1 kPa (7.0%) and 37.8 kPa (10.5%), respectively (with significance in studies I, II and IV). With respect to the heat pain assessment of the non‐irradiated area, the LSM (estimate of the difference) in studies I, II, III and IV was 45.1°C (4.1%), 47.3°C (0.6%), 47.1°C (0.7%) and 47.0°C (1.4%), respectively (with significance in studies I and IV). Compared to placebo, ibuprofen had a significant on the PDT in the heat pain assessment in all three studies in which ibuprofen was administered, with an LSM (estimate of the difference) in studies I, III and IV of 40.2°C (4.0%), 42.1°C (3.4%) and 41.8°C (3.1%), respectively. Similar results were obtained with respect to the other pain models.

**Table 2:** Significant clusters of the pharmacodynamic measurements

	Study I			Study II			Study III			Study IV		
	CV (%)	*p*‐value	LSMean effect versus placebo (95% CI)	CV (%)	*p*‐value	LSMean effect versus placebo (90% CI)	CV (%)	*p*‐value	LSMean effect versus placebo (90% CI)	CV (%)	*p*‐value	LSMean effect versus placebo (90% CI)
Pregabalin												
Electrical stimulation PTT	18.8	**0.0121**	1.46 [1.02; 1.20]	22.8	0.3697	1.31 [0.96; 1.15]	22.1	0.0701	1.22 [1.01; 1.19]	14.8	0.3489	1.22 [0.98; 1.09]
Pressure stimulation PTT	25.0	**0.0052**	1.13 [1.04; 1.25]	21.1	**0.0085**	1.05 [1.06; 1.26]	22.1	0.1420	1.09 [0.99; 1.15]	19.8	**0.0124**	1.03 [1.04; 1.18]
Cold pressor PTT	28.9	**<0.0001**	1.14 [1.27; 1.69]	30.6	**0.0005**	1.15 [1.16; 1.48]	26.6	**0.0008**	1.09 [1.11; 1.34]	19.8	**<0.0001**	1.10 [1.14; 1.32]
Heat pain PDT (UVB treated skin)	4.2	0.2671	4.10% [0.99; 1.03]	4.2	0.4483	0.21 [−0.45; 1.22]	3.3	0.1138	0.35 [−0.02; 0.95]	3.4	**0.0380**	0.63 [0.11; 0.96]
Heat pain PDT (normal [non‐treated] skin)	4.6	**0.0049**	1.20% [1.01; 1.07]	3.5	0.5705	0.38 [−0.41; 0.84]	2.8	0.2121	0.45 [−0.11; 0.81]	2.4	**0.0009**	0.53 [0.32; 0.93]
Ibuprofen												
Electrical stimulation PTT	19.0	0.7525	1.03 [0.94; 1.09]	–	–	–	18.0	0.5835	1.05 [0.95; 1.11]	14.5	0.5974	1.08 [0.93; 1.04]
Pressure stimulation PTT	25.4	0.2576	1.01 [0.96; 1.15]	–	–	–	18.0	0.1589	1.03 [0.99; 1.14]	19.4	0.0603	0.98 [1.01; 1.15]
Cold pressor PTT	28.9	0.6850	1.05 [0.89; 1.19]	–	–	–	22.6	0.4250	1.06 [0.95; 1.15]	19.4	0.1007	1.08 [1.00; 1.17]
Heat pain PDT (UVB treated skin)	4.5	**0.0006**	1.70% [1.02; 1.06]	–	–	–	2.8	**<0.0001**	−0.13 [0.91; 1.87]	3.4	**<0.0001**	0.18 [0.82; 1.70]
Heat pain PDT (normal [non‐treated] skin)	4.7	0.2080	4.00% [0.99; 1.05]	–	–	–	2.7	0.6191	1.39 [−0.57; 0.31]	2.2	0.3383	1.26 [−0.13; 0.50]

Note

CI: confidence interval; CV: inter‐individual coefficient variability; LSMean: least squares means; PDT: pain detection threshold; PTT: pain tolerance threshold; UVB: ultra violet B radiation. Average effects compared with placebo: study I: up to 5 hr post‐dose for pregabalin and ibuprofen; study II–IVL pregabalin up to 6 hr post‐dose, ibuprofen up to 4 hr post‐dose. Contrasts were calculated within the repeated measures mixed model. Significant values shown in bold. Confidence interval LOG transformed, heat pain (UVB and normal skin) in study II–IV are back‐transformed.

The coefficient of variance (CV), which reflects inter‐subject variability, was also analysed for both pregabalin and ibuprofen for each pain model separately in each study. With respect to pregabalin, the heat pain assessment of both the UVB‐irradiated skin and the non‐irradiated skin had the lowest variability, with all CV values <5%. The CV values for the electrical and pressure stimulation assessments were approximately 20%, and the cold pressor assessment had the highest CV values, ranging from 19.8% to 30.6%. Similar results were obtained for ibuprofen; specifically, the CV values for the heat pain assessment were <5%, both electrical and pressure stimulation had CV values ranging from 14.5% to 25.4%, and highest variation was measured for the cold pressor assessment, with CV values ranging from 19.4% to 28.9%.

**Figure 1:**
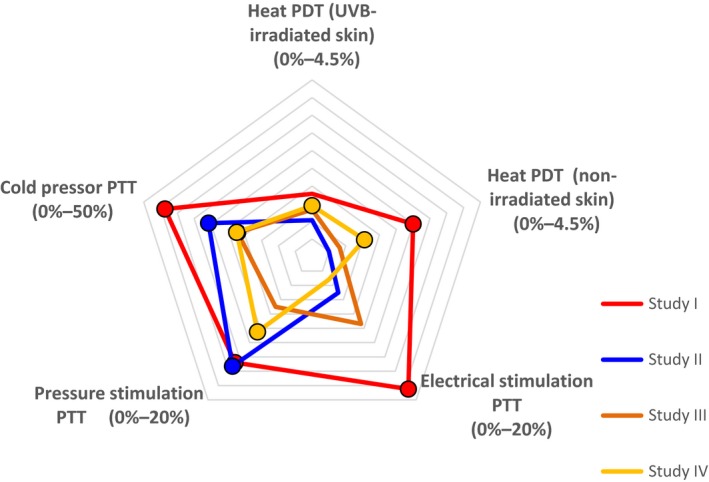
Star plot summarizing the effects of pregabalin (300 mg) on the indicated pain assessments in studies I through IV. The percentages shown for each assessment reflect the difference between pregabalin and placebo. Values marked with a circle are significantly different (*p* < 0.05) compared with placebo. PDT, pain detection threshold; PTT, pain tolerance threshold

**Figure 2:**
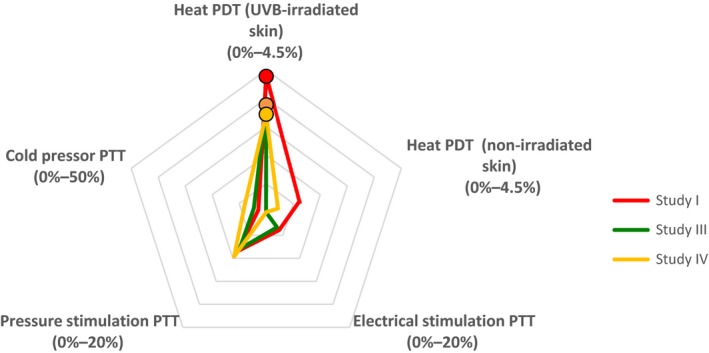
Star plot summarizing the effects of ibuprofen (600 mg) on the indicated pain assessments in studies I, III, and IV. The percentages shown for each assessment reflect the difference between ibuprofen and placebo. Values marked with a circle are significantly different (*p* < 0.05) compared with placebo. PDT, pain detection threshold; PTT, pain tolerance threshold

Figure 1 shows the profile of pregabalin based on the battery of pain models up to 6 hr after dosing (or up to 5 hr after dosing in the case of study I). Overall, the cold pressor and pressure pain models were most sensitive to the effects of pregabalin, whereas the heat and electrical models were the least sensitive to pregabalin. The electrical, cold pressor and heat pain in non‐irradiated skin models were all more sensitive to pregabalin in study I compared to studies II, III and IV. The effects of ibuprofen analysed up to 4 hr after dosing were similar to the effects of pregabalin; the profile of ibuprofen based on the battery of pain models is shown in Figure 2. All three studies with ibuprofen yielded a similar profile.

## 4 DISCUSSION

Here, we report that using a battery of five pain models yielded highly consistent results with respect to the qualitative and quantitative analgesic effects of both pregabalin and ibuprofen. This high reproducibility means that obtaining a profile for a given drug using this battery of pain tests can reveal the “trait” of that drug. Our results demonstrate that inter‐subject variability is relatively low with respect to assessing heat‐induced pain in both UVB‐irradiated and non‐irradiated skin; thus, we found an overall consistent level of efficacy for two commonly used analgesics compared to placebo. Importantly, ibuprofen had a significant analgesic effect in all three studies that used this drug, suggesting that the UVB‐induced heat pain model is highly sensitive to this class of compounds. This finding is consistent with expected pharmacodynamic outcomes using this pain model, as the UVB model is considered a suitable model for inflammatory pain and is therefore highly sensitive to nonsteroidal anti‐inflammatory drugs (NSAIDs; van Amerongen et al., 2016; Bishop et al., 2009). The lack of an effect of ibuprofen in the other pain models was also highly consistent, supporting the high reproducibility of these models. The lack of an effect with ibuprofen with the other pain models can be explained by the fact that these models are not based on inflammation‐related hyperalgesia, but rather are mediated by acute nociceptive pain. The fact that the PD profile for ibuprofen differs from the PD profile for pregabalin indicates that specific compounds can produce a highly specific profile in a battery of pain models; moreover, we recommend than several modalities be included in the design of future studies in order to obtain a more complete profile of the test compound. Such an approach will likely yield important information that can be used to make a more informed decision regarding the next phases in the drug development process.

With respect to pregabalin, a wide range of pain models has been used to demonstrate significant analgesic effects, and pregabalin has anxiolytic, anticonvulsant and analgesic properties (Beydoun et al., 2005; Elger, Brodie, Anhut, Lee, & Barrett, 2005; Feltner et al., 2003; Hill et al., 2001; Morera‐Domínguez, Ceberio‐Balda, Flórez‐García, Masramón, & López‐Gómez, 2010; Pande et al., 2003; Rickels et al., 2005; Satoh et al., 2010). Pregabalin is a structural analogue of the inhibitory neurotransmitter gamma‐aminobutyric acid (GABA) and binds to the α_2_δ auxiliary subunit of voltage‐gated calcium channels in the central nervous system. In clinical trials, pregabalin has been shown to have analgesic effects in diabetic neuropathy, post‐herpetic neuralgia and spinal cord injury (Offord & Isom, 2016; Patel & Dickenson, 2016; Tatji et al., 2016). In controlled clinical trials involving peripheral neuropathic pain, 35% of pregabalin‐treated patients had an improvement in their pain scores (Satoh et al., 2010). It is important to note, however, that pregabalin has also been shown to provide analgesic effects in nociceptive pain, including dental patients following extraction of the third molar (Hill et al., 2001) and in patients suffering from lower back pain (Morera‐Domínguez et al., 2010). This broad analgesic efficacy is reflected in the profile shown in Figure 1, in which the majority of pain models were sensitive to pregabalin. This finding suggests that combining these well‐established pain models—which together represent distinct, complementary mechanisms—can have high predictive value with respect to the efficacy of an analgesic in clinical practice.

Using our battery of evoked pain models, we were able to detect differences in both pharmacological and analgesic properties. Both ibuprofen and pregabalin produced a unique profile of analgesic effects in pain evoked using the models included in these studies. Pregabalin is known to induce somnolence, which can affect its analgesic outcome. We recently reported that this battery of pain models is not affected by sedation, as the classic H1 antihistamine promethazine served as a negative control (van Amerongen, Siebenga, Kam, Hay, & Groeneveld, 2018). Most of the analgesic effects that we observed for ibuprofen and pregabalin are consistent with previous reports and with their expected PD and PK profiles, and both compounds have been shown to provide analgesic effects in clinical practice in nociceptive and/or neuropathic pain at the doses used in our studies. The analgesic profile that drugs exhibit in this multimodal pain test setting in healthy subjects may possibly be linked in the future to the subgroups of patients with neuropathic pain—divided based on phenotypical differences tested with e.g., Quantitative Somatosensory Test—who appear to respond better to certain treatments than other phenotypical subgroups, but this needs further exploration (Demant et al., 2014; Holbech et al., 2016).

Inconsistencies that emerge from a reproducibility study do not necessarily indicate that the method is unreliable. For example, the profile for pregabalin in study I differed from the profiles obtained in the other three studies (Figure 1); however, this difference was likely due to the use of a different formulation of pregabalin in the first study. The dissolution and absorption of pregabalin could have been altered by this different formulation, which could have affected the plasma concentration over time. Additional analyses of the PK profiles among the various studies revealed that the systemic exposure of pregabalin was higher in study I than in the other three studies. Importantly, additional PK/PD model‐based analyses of each endpoint and the concentration–response curves increased reproducibility by accounting for differences in exposure between subjects and studies (data not shown). Although this additional analysis confounded the reproducibility by dose, it provided direct insight into the test battery's sensitivity to differences in the plasma concentration of an analgesic compound, and it demonstrated reliability by concentration. The electrical stimulation assessment, the cold pressor assessment in particular and—to a lesser extent—the heat pain assessment of non‐irradiated skin were affected more strongly by pregabalin in the first study than in the other three studies. This suggests that the maximum effective concentration of pregabalin may not have been reached in studies II–IV and that this battery of pain models can be used to distinguish between dose‐dependent and concentration‐dependent effects of pregabalin. Thus, using ascending doses may improve the profile of a drug's analgesic effects, and testing more than one dose of a compound in the battery of pain models will likely provide a more complete overview of the compound's analgesic potential. Another factor that may have contributed to the difference in outcome between study I and the other three studies is that study I included both male and female subjects, whereas the other three studies were restricted to male subjects only (Okkerse et al., 2017).

When using pain models to assess the putative analgesic properties of a drug, it is important to determine the precision of the measurements obtained with these pain models. Unfortunately, however, the reproducibility of major findings published in high‐profile journals is strikingly low, ranging from 10% to 25% (Peers, Ceuppens, & Harbron, 2012; Prinz et al., 2011). Thus, increasing the reproducibility of a method is an important step in the scientific method, allowing science to progress by building on previous research. Achieving this goal requires the submission of both the data and the computational and analytical tools that were used to generate the results; without this information, the results cannot be verified and built upon. Adherence to established guidelines regarding the conduct of experimental research is also important, as is proving access to the protocol and the data collected (Begley & Ioannidis, 2015). On the other hand, a single well‐defined scientific method that results in a constructive scientific process is—at best—debatable. Additional submission of the data may lead to mistrust among researchers and possible over‐regulation with respect to the acceptance of manuscripts based on seemingly narrow technical criteria. Misconduct has always been a part of science, with surprisingly few consequences. Distrust by the public is likely higher thanks to the apparent variability among scientific conclusions (Drummond, 2018). Consensus regarding how this variability can be addressed is unlikely. A combination of approaches has been used, including an assessment of test–retest reliability, intraclass correlation coefficient and the level of agreement (Cathcart & Pritchard, 2006; Geber et al., 2011; Gelber et al., 1995; Nothnagel et al., 2017; Shy et al., 2003; Siao & Cros, 2003). Reliability analysis has also been used widely, generally yielding high reliability among results (Agostinho et al., 2009; Alappattu, Bishop, Bialosky, George, & Robinson, 2011; Geber et al., 2011; Gehling et al., 2016; Heldestad, Linder, Sellersjo, & Nordh, 2010; Knutti, Suter, & Opsommer, 2014; Lowenstein, Jesse, & Kenton, 2008; Moloney, Hall, & Doody, 2012; Moloney, Hall, O’Sullivan, & Doody, 2011; Nothnagel et al., 2017; Wylde, Palmer, Learmonth, & Dieppe, 2011). The aim of our study was to measure consistency of the profile of two analgesic drugs using our pain test battery. When the drug profile is consistent among populations, the profile can be regarded a “trait”—in other words, a pharmacological biomarker. Our approach revealed consistently reproducible results with respect to the analgesic profiles of both pregabalin and ibuprofen in a heterogeneous study population, suggesting that variability regarding the perception of pain among subjects likely plays only a small role. As discussed above, using the appropriate study design for each compound under investigation is particularly important.

Our finding that the results obtained using our battery of pain models are reproducible—thus yielding a reliable profile of analgesic effects when testing different compounds—supports the notion that this test battery can be used reliably in the early stages of clinical drug development. For example, this battery of tests can be used to screen drugs for their analgesic potential and/or to determine the analgesic dose/concentration range of new analgesic compounds in the early stages of clinical development. Importantly, creating an extensive database containing the profiles of established analgesic compounds can provide a series of benchmarks for comparing new compounds to existing analgesic drugs and can help researchers predict the efficacy of new compounds in specific patient populations.

## 5 CONCLUSIONS

Here, we report that four separate studies with a similar design and using a battery of evoked pain models involving healthy human subjects yielded highly reproducible results with a low CV. The consistency and the reproducibility of the analgesic profile at clinically effective doses validates the use of this pain test battery as a tool for demonstrating analgesic activity and for helping establish the optimal active dose in early clinical drug development.

## CONFLICTS OF INTEREST

None declared.

## AUTHOR CONTRIBUTIONS

PS, GA, PO, RB, JH and GG conceived and designed the study. PS, GA, PO, JH and GG collected the data. WD and PD analysed the data. PS, JH and GG wrote the manuscript, and GA, PO, WD, PD, and RB provided valuable comments regarding the manuscript. All authors have approved the submitted manuscript.
